# Testing the consistency of wildlife data types before combining them: the case of camera traps and telemetry

**DOI:** 10.1002/ece3.997

**Published:** 2014-02-24

**Authors:** Viorel D Popescu, Perry Valpine, Rick A Sweitzer

**Affiliations:** 1 Department of Environmental Science, Policy and Management, University of California Berkeley130 Mulford Hall #3114, Berkeley, California, 94720-3114

**Keywords:** Camera trap, capture rate, data consistency, detection probability, fisher, home range, *Pekania pennanti*, Sierra Nevada, telemetry, wildlife monitoring

## Abstract

Wildlife data gathered by different monitoring techniques are often combined to estimate animal density. However, methods to check whether different types of data provide consistent information (i.e., can information from one data type be used to predict responses in the other?) before combining them are lacking. We used generalized linear models and generalized linear mixed-effects models to relate camera trap probabilities for marked animals to independent space use from telemetry relocations using 2 years of data for fishers (*Pekania pennanti*) as a case study. We evaluated (1) camera trap efficacy by estimating how camera detection probabilities are related to nearby telemetry relocations and (2) whether home range utilization density estimated from telemetry data adequately predicts camera detection probabilities, which would indicate consistency of the two data types. The number of telemetry relocations within 250 and 500 m from camera traps predicted detection probability well. For the same number of relocations, females were more likely to be detected during the first year. During the second year, all fishers were more likely to be detected during the fall/winter season. Models predicting camera detection probability and photo counts solely from telemetry utilization density had the best or nearly best Akaike Information Criterion (AIC), suggesting that telemetry and camera traps provide consistent information on space use. Given the same utilization density, males were more likely to be photo-captured due to larger home ranges and higher movement rates. Although methods that combine data types (spatially explicit capture–recapture) make simple assumptions about home range shapes, it is reasonable to conclude that in our case, camera trap data do reflect space use in a manner consistent with telemetry data. However, differences between the 2 years of data suggest that camera efficacy is not fully consistent across ecological conditions and make the case for integrating other sources of space-use data.

## Introduction

Making use of complementary information from different data sources is a common theme in recent advances in statistical modeling in ecology. Examples include combining marked individual data and population surveys to estimate integrated population models (Besbeas et al. 2002; Johnson et al. 2010), and combining camera trap data and fecal DNA data (Gopalaswamy et al. 2012) or mark-resight and telemetry data (Ivan et al. 2013; Royle et al. 2013; Sollmann et al. 2013a,b2013b) to estimate animal density using spatial capture–recapture models.

Estimating abundances and demographic parameters from camera traps has become prominent in wildlife research in the past two decades (Karanth and Nichols 1998). However, estimating abundance from camera trap grids alone is difficult if the movement area of animals is unknown. Recently developed spatial capture–recapture models for camera traps with marked animals allow estimation of activity centers under simple assumptions of home range shapes and detection probabilities (e.g., animals have isotropic home ranges, and camera detection probabilities are a function of the Euclidean distance between camera traps and individual activity centers; Efford 2004; Royle and Young 2008). Incorporating telemetry data into the analysis can allow stronger information about space use to provide better animal abundance (Ivan et al. 2013; Sollmann et al. 2013a,b2013b), as well as landscape connectivity estimates (Royle et al. 2013).

However, what these and other efforts to combine data types have in common is the assumption that different data types are inherently consistent with each other (i.e., different types of data are measuring the same quantities, without any biases relative to each other). This assumption has gone largely untested and, in general, it should not be taken for granted. For example, camera traps are often baited, which may impact animal space use during the camera trap deployment. Camera data and telemetry data may be obtained at different times of day, across different seasons or sampling windows, or may be impacted by different explanatory variables. In addition, heterogeneity between animals and/or cameras in detection probability may be difficult or impossible to estimate from camera trap data alone. Thus, there is a need for investigating how different data types are related and testing whether they can be combined without additional assumptions. In this study, we take the approach of holding camera trap capture and telemetry datasets apart from the each other, and asking whether information from one can predict responses in the other.

**Figure 1:**
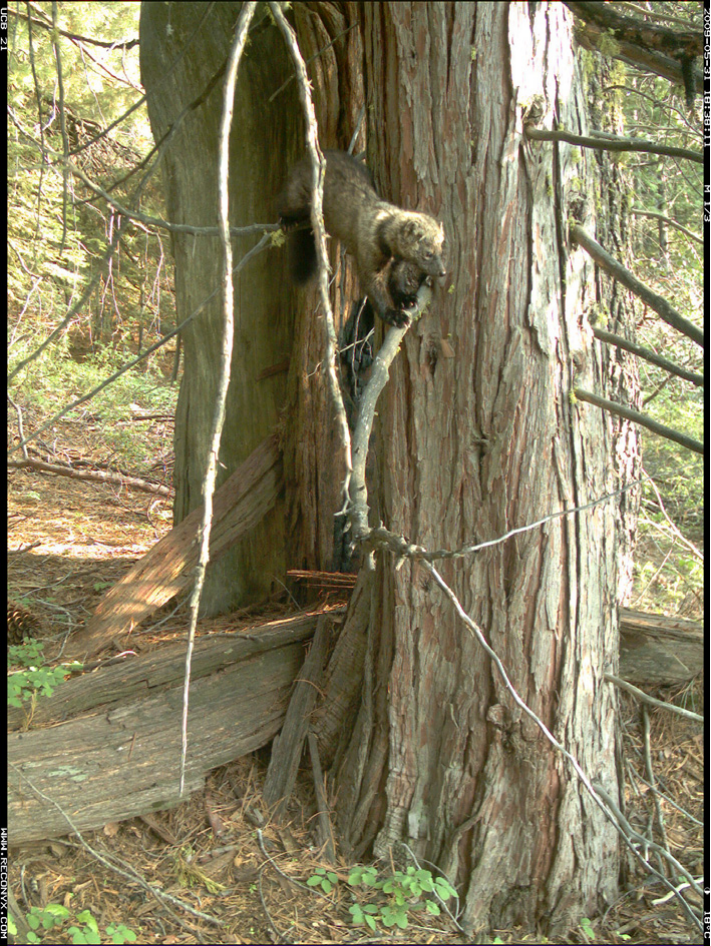
Adult female fisher (*Pekania pennanti*) photographed near a den tree in the Sierra Nevada Mountains, California USA.

In our study system, extensive camera trap and telemetry data were gathered simultaneously for fishers (*Pekania pennanti*; Fig. 1), a forest carnivore that is now uncommon and rare in the western part of its range in North America (Lewis et al. 2012). We asked two specific questions about the relationship between these types of data: (1) how does the number of telemetry relocations near camera traps predict camera detection probability, including differences between sexes and seasons (*Proximity analysis*)? and (2) does home range utilization density (space-use frequency) estimated from telemetry data adequately predict camera detection probability (*Home range analysis*)? The first objective provides insight into camera trap efficacy that is directly interpretable in terms of animal activity near traps, which may be useful for determining performance of the trapping grid in terms of spacing and layout. The second objective evaluates whether camera traps measure space use consistently to telemetry.

For the first objective, we considered several hypotheses about factors that might shape the relationship between animal activity near a camera trap and detection at that trap. Male fishers have significantly larger home ranges than females and perform more frequent movements in search of mates and to defend territories (Zhao et al. 2012); thus, males will likely have access to more camera traps within home ranges. If males and females have a similar propensity to visit camera traps, we hypothesized that males are more likely to be detected at camera traps compared with females given the same number of nearby telemetry relocations. Because the camera traps were baited using a combination of scent lures and venison, we also hypothesize that detection probabilities would be higher during the winter season, when food is scarcer. Finally, we hypothesized that there may be heterogeneity among animals in their propensity to be detected due to differences in their ability to discover camera traps and/or habituation to specific traps.

For the second objective, we hypothesized that the utilization distribution should predict camera trap detection probability, possibly with differences between sexes. Alternatively, if animals visit camera stations out of proportion to predictions from home range space use, that pattern could indicate that traps induce fundamentally different movement behaviors (e.g., avoidance or attraction to camera trap stations). For this objective, we chose a set of models including the utilization distribution along with other possible factors and performed model selection using Akaike Information Criterion (AIC), AIC_c_, and QAIC. In general, because animals spend more time in the core areas of their home ranges, they may be disproportionately likely to find a camera trap there. In other words, animals may know their core area in more detail than outlying areas and hence they may encounter camera traps in the core area more often than would be expected, simply based on its size and the proportion of time spent there. We evaluated several models representing this idea using either a categorical variable for core/noncore areas or a continuous variable for home range isopleths, as candidate variables for more complex models to explain camera trap detections. We also considered models with nonlinear relationships between utilization density and detection probability. Finally, we considered heterogeneity between animals in their propensity to be detected by cameras and/or between individual cameras in their propensity to attract animals.

## Materials and Methods

### Study area

The study area was the nonwilderness region of the Bass Lake Ranger District in the Sierra National Forest, near Oakhurst, California, and covered approximately 1150 km^2^. This area is topographically complex with elevations ranging from 758 m to 2652 m. Primary tree species include incense cedar (*Calocedrus decurrens*), white fir (*Abies concolor*), ponderosa pine (*Pinus ponderosa*), sugar pine (*Pinus lambertiana*), giant sequoia (*Sequoiadendro giganteum*), black oak (*Quercus kelloggii*), and live oak (*Quercus* spp.). The study area is part of the larger project SNAMP, the Sierra Nevada Adaptive Management Project, which was formed to evaluate the impact of strategically placed forest fuel treatments on wildlife (specifically the California spotted owl, *Strix occidentalis occidentalis,* and the fisher), water resources, forest health, and fire prevention.

### Trapping and radio telemetry

To obtain animals for telemetry, individual fishers were live-captured in steel mesh traps (Tomahawk Live Trap Company, Tomahawk, WI) with a plywood cubby box to provide shelter. Trapping was focused during the fall and winter seasons between October 2007 and September 2011. Each animal was fitted with a radio-collar (Holohil Systems Model MI-2M, Ontario, Canada; Advanced Telemetry Systems Model 1930 or 1940, Isanti, MN). All radio collars were modified by attaching small bands (0.5–1.0 cm) of infrared reflective tape (3M® Scotchlite™, St. Paul, MN) along the lengths of the antennas, which were used to identify individual radio-collared fishers detected at camera traps. Radio-collared fishers were subsequently monitored and relocated 4–6 days/week throughout the year by fixed-wing airplane (Thompson et al. 2012). We assessed the accuracy or error associated with aerial telemetry locations by calculating the distance between the estimated locations obtained by the biologist in the airplane and known locations of “test” collars at fixed positions (*n* = 501 test collars). Mean error was estimated at 338.9 m (range = 14.6–1219.8 m).

For this analysis, we only used 2 years of telemetry data from the larger study: October 1, 2008–September 30, 2009 (Year 1) and October 1, 2009–September 30, 2010 (Year 2). During this period, 52 fishers (32 females and 20 males) were radio-tracked, of which 19 females and 7 males were tracked during both years.

### Camera trap deployment

Automatic cameras (Silent Image Professional and Rapidfire PC85 series; RECONYX Inc., Holmen, WI) were systematically deployed near the center of 1 × 1-km grid cells overlain on the study area (Year 1 = 341 locations; Year 2 = 403 locations). It was not possible to survey all 1-km^2^-grid cells at the same time; the maximum number of cameras deployed at one time was 60 (Year 1) and 85 (Year 2), and the patterns of camera deployment were closely related to ease of access (e.g., snowpack during winter). Placement of camera stations within 1-km^2^-grid cells was determined based on the presence of habitat elements known important for fishers including presence of mature or large diameter trees, moderate to steep slopes, relatively high canopy cover (≥60%), and proximity to permanent streams (Zielinski et al. 2004).

Camera trap stations were baited with a combination of meat and scent lures. On each bait tree, the following were attached: (1) a dark colored sock stuffed with venison (140–250 g) and (2) 8–10 hard-shell pecans strung onto wire. Peanut butter and Hawbaker's Fisher Scent Lure (Fort Loudon, PA) were smeared on the nut ring and bait sock, respectively, and Caven's “Gusto” long distance call lure (Minnesota Trapline Products, Pennock, MN) was applied near the base of the bait trees and on several nearby trees. Camera survey stations were visited every 8–10 days over 32–40 days to refresh scent lures and bait and to maintain camera units (Slauson et al. 2009).

Detections of collared fishers were extracted from images based on the antennal pattern of bands of infrared reflective tape. Camera detections of fishers were identified based on groups of fisher images separated by at least 15 min, and not all radio-collared fishers detected at camera survey stations could be unambiguously identified due to occasional loss of bands and breakage/loss of collar antennas.

### Home ranges

We built annual (October–September) and seasonal (*Fall/Winter*: 1 October–15 March, and *Summer*: 1 June–30 September) home ranges for each individual fisher using the fixed kernel density method in Home Range Tools for ArcGIS 9.3 (Rodgers et al. 2007). We omitted the period 16 March–31 May from the seasonal home ranges because it roughly encompasses the reproductive season when males expand their movements in search of reproductive females, and reproductive females concentrate their movements around tree den used to produce and nurture their offspring (Weir et al. 2012). For each individual home range, we extracted the isopleth (to 1% accuracy) and utilization density for each camera location. For all analyses, we only used home range data up to the 90% isopleth because few locations occurred beyond this region and kernel smoothing is less accurate in the tails. Using the reference bandwidth tends to oversmooth the home range contours (Wand and Jones 1995), so we manually selected the bandwidths among values of 0.6, 0.7, 0.8, 0.9 times the reference bandwidth. The main criteria for bandwidth selection were isopleth interval cohesion (intervals did not break up into small polygons), and the extent of the 90th and 95th isopleth (isopleths did not extend well beyond the location data extremes). In most cases, the bandwidths chosen were within the 0.6–0.9 range, with 2 (1.7%) seasonal home ranges and 20 (25%) yearly home ranges being assigned the reference (1.0) bandwidth. Sample sizes for estimating annual and seasonal home ranges were large, and we expect high accuracy when using our method for bandwidth selection. The median number of relocations used for annual home range estimation was 164 (range = 37–276), and for the seasonal home ranges was 84 (range = 25–155).

### Methods for comparing telemetry and camera trap data

#### Camera trap availability

We defined as “available” each camera trap that had the potential to capture a collared fisher photo based on its location and time of deployment. We first identified the locations of camera traps within home ranges using the 90% isopleth as the maximum extent. Second, we determined which camera traps were available for each collared fisher temporally by examining the overlap between the camera trap deployment windows and the period each fisher was actively radio-tracked (e.g., to exclude mortality or dropped/inactive collar events). Similar to the home range analyses, we omitted camera trap data collected during the reproductive season.

#### Proximity analysis

For each collared fisher and available camera, we extracted the number of telemetry locations within 250 and 500 m circular neighborhoods centered on camera trap locations (*NLocs*). We limited the extent to 500 m to ensure that each telemetry location was only counted for one camera trap; cameras were located 1000–1400 m from each other (e.g., within 1-km^2^ grids); thus, the maximum of 500 m ensured that each telemetry location could only be counted once. We used variable *Season* to test for differences in detection probability between periods with high (*Summer:* June–September) and low (*Fall/Winter:* October–March) food availability.

We used generalized linear mixed-effects models (GLMM; McCulloch et al. 2008) to investigate whether the number of known telemetry locations within 250 and 500 m from camera traps can be used to predict the camera capture probability of collared animals (Year 1: 35 fishers, 208 camera locations, and 547 fisher/camera combinations; Year 2: 35 fishers, 212 camera locations, and 371 fisher/camera combinations). We used a binary response variable indicating detection [1] (e.g., fisher was photographed at least once at a given camera trap) or nondetection [0], and ran models with the number of telemetry locations (*NLocs*), sampling season (*Season*), and *Sex* as fixed effects, and individual *Fisher* as a random effect. Along with additive models, the candidate model set contained the interaction terms *Sex* × *Season* and *NLocs* × *Sex*. We used Akaike Information Criterion corrected (AIC_c_ for small sample size) for model selection and likelihood ratio tests to examine a priori hypotheses (Royle and Dorazio 2008).

#### Home range analysis

According to the theory behind kernel density estimation of home ranges, the utilization distribution describes the estimated frequency of space use at any location. The probability that an animal is found in a small area is proportional to that area times the utilization density (*UD*) at that location, and the entire surface of utilization densities is the utilization distribution. Therefore, one would predict that probability of detection at a camera trap should be proportional to the *UD* at the camera location, which we call the *simple model*. We expect that the proportionality constant could differ between males and females for behavioral reasons, with males moving across larger areas and thus more likely to find cameras, but potentially spending less time in areas surrounding any one camera.

Alternatively, if camera trap probabilities are not predicted solely by *UD* at camera locations, then additional variables and/or model forms will provide better predictions. For example, highly heterogeneous home range sizes would yield utilization densities not perfectly correlated to the isopleth percentile. First, if including isopleth percentiles themselves (*Isopleth,* to 1% accuracy) or a categorical variable for the core versus noncore parts of the home range (*Core*), separated by the 50% isopleth, provides a better model, it would mean animals tend to find cameras in the central versus peripheral parts of their home range more or less often relative to the time they spend in those areas. Second, we considered that probability of finding a camera could vary nonlinearly with *UD*, meaning that larger or smaller isopleth values lead to different photo probabilities beyond just the effect of *UD*. Third, we considered interactions between these variables and *Sex*.

We considered two types of response variables: (1) a binary response indicating whether each available camera ever captured a photo of each animal and (2) a count response indicating the number of times each camera detected each animal. The binary variable allows modeling of camera trap probabilities without complications due to behaviors induced by camera traps themselves (e.g., “trap-happiness” due to baiting or “trap-shyness”), or other latent factors. The count variable uses more information from the cameras, but at the cost of these additional complications for interpretation.

A set of candidate models was represented by generalized linear (possibly mixed-effects) models (GLM or GLMM). For the binary response, we used a complementary log-log (*cloglog*) link and binomial or quasibinomial variation. The *cloglog* link is more appropriate for modeling detection/nondetection as a spatial process, as opposed to the more traditional logit link approach (Baddeley et al. 2010). For count responses, we used a log link with Poisson or quasipoisson variation. Consider a GLM with the linear part describing the log rate of camera captures:


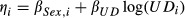
(1)

Here, *β*_*Sex,i*_ takes one value if the animal in observation *i* is male and another if it is female; log(*UD*_*i*_) is the natural log of the utilization density of the camera for observation *i*; and *β*_UD_ is a coefficient for log(*UD*_*i*_). The right-hand side can be extended to other combinations of fixed and random effects.

The rate of camera captures is:


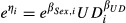
(2)

Thus, exp[*β*_*Sex,i*_] is the *slope* for the utilization density. And if the simple model is correct, *β*_UD_ should be 1. In some candidate models, we estimate *β*_UD_ to see whether it deviates from 1, while in others, we set it to 1. Setting it to 1 means that the value of 1*log(*UD*_*i*_) is forced into each linear predictor (equation 1), which is called an *offset*. Thus, our *simple model* is denoted as (*Sex + offset*[*log*(*UD*)]).

For count responses, equation (2) gives the expected value. For binary responses, the *cloglog* link gives the probability of at least one camera capture over a fixed time interval as


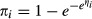
(3)

The *cloglog* link itself is *η*_*i*_* = log*(*−log*[*1−π*_*i*_]).

For each type of response variable, we compared a predefined set of hypotheses that included the simple model (*Sex + offset*[*log*(*UD*)]), as well as nonlinear effects of *UD* (i.e., *β*_UD_ estimated) and additive and interactive effects of *Isopleth* or *Core*, with or without *UD*. We evaluated these models using model selection with AIC (GLMMs), AICc (GLMs), or QAICc (GLMs with quasilikelihoods; Burnham and Anderson 2002). The GLMMs and quasilikelihoods represent two different ways to accommodate overdispersion (Fieberg et al. 2009). For the GLMMs, we first selected random effects using models with saturated fixed effects and then used the chosen random effect structure to compare different fixed effects (Zuur et al. 2009). We used *Fisher* as a random effect (*n* = 26 fishers in Year 1 and *n* = 18 fishers in Year 2) to account for behavioral heterogeneity between animals [1 | Fisher]. We also examined the use of random effects for *Camera* [1 | Camera], and *Camera* × *Fisher* combinations [1 | Fisher/Camera], to account for unexplained heterogeneity related to camera location only, and camera location within a fisher home range, respectively (Year 1: 131 camera trap locations, 402 fisher/camera combinations; Year 2: 123 camera trap locations, 330 fisher/camera combinations). GLMM fitting was performed using package *lme4* (Bates et al. 2012) for R 3.0.1 (R Core Team 2013), and we used package *AICcmodavg* (Mazerolle 2012) for model selection.

#### Examining the relation between the proximity-to-cameras and home range analyses

The two analyses used different information from the same dataset, but were ultimately used to predict the same quantity: probability of detection at camera traps. The proximity analysis did not use home range information, while the home range analysis did not account for the distance of relocations from cameras. To examine how these analyses fit together, we visually investigated the relation between (1) the number of telemetry relocations for those cameras that were successful at detecting fishers and (2) the utilization density at which these cameras were located.

## Results

### Fisher home ranges

Across both years, male annual home ranges (95% kernel density estimates) were greater than female home ranges (8915.8 ± 963.5 ha vs. 2910.0 ± 416.7 ha for males and females, respectively; Mann–Whitney *U *=* *84.0, 1 d.f., *P*-value < 0.0001; Table 1). *Fall/Winter* home ranges were greater than *Summer* home ranges for females (2490.9 ± 382.4 ha vs. 1310.4 ± 254.2 ha; Mann–Whitney *U *= 437.0, 1 d.f., *P-*value < 0.0001), and also for males, although the result was not statistically significant (6049.8 ± 497.1 ha vs. 4867.3 ± 323.6; Mann–Whitney *U *=* *64.0, 1 d.f., *P*-value = 0.263; Table 1).

**Table 1:** Mean (±1 SE) annual and seasonal home range sizes of fishers estimated using kernel density methods (data from both years combined); *n* = number of individuals

		Males		Females	
Home range type	Period	Area (ha)	*n*	Area (ha)	*n*
Annual	1 October–30 September	8915.8 ± 963.5	23	2910.0 ± 416.7	43
*Fall/Winter*	1 October–15 March	6049.8 ± 497.1	14	2490.9 ± 382.4	30
*Summer*	1 June–30 September	4867.3 ± 323.6	7	1310.4 ± 254.2	18

### Proximity analysis

**Figure 2:**
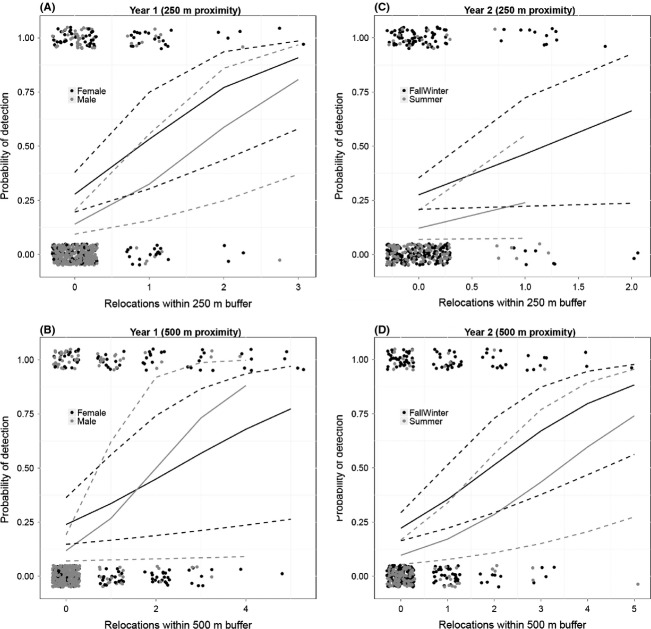
Predicted probability of detection at camera traps of male and female fishers based on telemetry relocations within <250 m and <500 m from camera traps during Year 1 (A and B) and Year 2 (C and D), based on the best generalized linear mixed-effects models with a random effect for each fisher ([1 Fisher]) for each year and distance (see Table 2). Dots represent vertically and horizontally jittered binary detection/nondetection data. The solid lines are mean probability calculated using fixed effects only; dotted lines are 95% confidence intervals.

During both years, there was a positive relationship between the number of telemetry relocations within 0–250 and 0–500 m buffers and the probability of an animal being detected at camera traps, and there were differences between years based on *Season* and *Sex* (Fig. 2).

During Year 1, the best models yielded differences in detection probability between males and females (given the same number of relocations), but not between seasons (Fig. 2A, Table 2; see Appendix S1 in Supplementary Information for full set of models). Detectability based on relocations within 250 m from camera traps was consistently higher for females across the entire range of relocations (Fig. 2A). The best model for the 500 m data included the interaction *Sex* × *Season* × *Locs500* (Table 2). The mean probability of detection for females increased from 0.33 (*n* = 1 relocation) to 0.77 (*n* = 5 relocations; Fig. 2B), while male detectability increased sharply, and required only three relocations to reach a 0.73 detection probability (Fig. 2B).

**Table 2:** Proximity analysis results using binary generalized linear mixed-effects models (GLMM; each model contains a random effect [intercept] for each fisher). Models within 2 AIC_c_ units of the top model are shown. 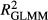 represents the conditional *R*^2^ for general linear mixed-effects models developed by Nakagawa and Schielzeth (2013)

Model	K	ΔAIC_c_	AIC_c_Wt	Cum.Wt	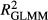
Year 1 – 250 m data					
*Sex × Season + Locs250*	6	0	0.39	0.39	0.24
*Sex + Season + Locs250*	5	0.83	0.26	0.65	0.20
Year 1 – 500 m data					
*Sex × Season × Locs500*	9	0	0.82	0.82	0.42
Year 2 – 250 m data					
*Season + Locs250*	4	0	0.48	0.48	0.13
*Season × Locs250*	5	1.53	0.22	0.7	0.13
*Sex + Season + Locs250*	5	1.79	0.19	0.9	0.14
Year 2 – 500 m data					
*Season + Locs500*	4	0	0.4	0.4	0.18
*Season × Locs500*	5	0.15	0.37	0.77	0.17

AIC, Akaike Information Criterion; K, number of parameters; AIC_c_Wt, AIC_c_ weight; Cum.Wt, cumulative AIC_c_ weight.

During Year 2, the simple *Season* models were best, and detection probability was consistently lower (by 0.2) during *Summer* compared to *Fall/Winter* for both buffer distances (Table 2, Fig. 2C and D). Sex-based differences in detection probability were not evident.

### Home range analysis

#### Binary data

**Figure 3:**
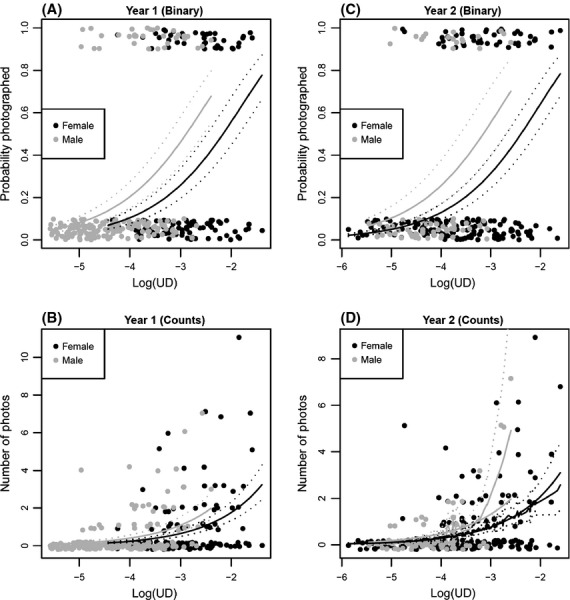
Estimated models relating camera trap data to log(*UD*) of home ranges. For binary data (A and C), the simple model was selected (*Sex + offset*[*log*(*UD*)]). Model predictions are shown with gray (male) and black (female) solid lines, with dotted lines for 95% confidence intervals. For count data (B and D), the simple model was either selected or almost selected. The count models shown are for the quasi-Poisson GLM method. In Year 2 (D), the simple model is shown as smooth solid lines, and the selected model [*Sex × Isopleth*] is shown as jagged solid lines with dotted 95% confidence intervals, indicating that three male camera counts in high use areas support the more complex model. Binary data are plotted with a random vertical jitter.

The best model using AIC_c_ for the binary camera data for both years was the simple model with photo probability proportional to *UD* (Table 3 and Appendix S2). The effect of *Sex* was significant, indicating that males are more likely than females to find a camera given the same intensity of space use as measured by telemetry-based *UD* estimates. In both cases, there are several models with small AICc differences from the best model. When the coefficients for any of these are considered with their confidence intervals, they include a linear relationship with respect to *UD* (*β*_UD_ not significantly different from 1, consistent with the simple model, Fig. 3A and C).

**Table 3:** Home range analysis results for *Fall/Winter* Year 1 for binomial models. Response variable is whether an available camera ever saw a particular animal. Models within 2 AIC_c_ units of the top model are shown, as well as best model that does not include log(*UD*). See Table S2.1 for complete list

Model	K	ΔAIC_c_	AIC_c_Wt	Cum.Wt
*Sex + offset(log[UD])*	2	0.00	0.18	0.18
*Sex × log(UD)*	4	0.25	0.16	0.35
*Sex × Core + offset(log[UD])*	4	0.85	0.12	0.47
*Sex × Isopleth + offset(log[UD])*	4	1.94	0.07	0.54
*Sex × Isopleth*	4	9.30	0.01	1.00

*UD*, utilization density; K, number of parameters; AIC_c_Wt, AIC_c_ weight; Cum.Wt, cumulative AIC_c_ weight.

Several “saturated” GLMM models were considered for selection of random effect terms, but none yielded better AIC for either year compared with binomial GLMs, so further binomial GLMMs were not considered.

#### Count data

For both years, count data models required accommodation of overdispersion (Table 4, Appendix S2). The simple GLM quasi-Poisson model (*Sex + offset*[*log*(*UD*)]) was the best model for Year 1 (Table 4, Appendix S2). For Year 2, there were three models with higher QAICc than the simple model, each including some effects of *Isopleth* and/or nonlinearity in *UD* (Appendix S2). These models suggest that fishers have a weakly supported tendency to visit cameras more frequently in the central part of a home range (i.e., lower *Isopleth* intervals). Moreover, the *Sex* × *Isopleth* interaction appears to be driven by few male camera pairs with high photo counts in low isopleth intervals (Fig. 3B and D).

**Table 4:** Home range analysis results for *Fall/Winter* Year 1 for count models. Response variable is the number of photos of a particular animal taken by an available camera. Models within 2 QAIC_c_ units of the top model are shown, as well as best model that does not include log(*UD*). Overdispersion parameter from saturated model was 2.50. See Table S2.3 for complete list

Model	K	ΔQAIC_c_	QAIC_c_Wt	Cum.Wt
*Sex + offset(log[UD])*	3	0.00	0.15	0.15
*Sex × Core + offset(log[UD])*	5	0.22	0.14	0.29
*Sex + Isopleth + offset(log[UD])*	4	1.23	0.08	0.38
*Sex × log(UD)*	5	1.30	0.08	0.46
*Sex + log(UD)*	4	1.52	0.07	0.53
*Sex + Core + offset(log[UD])*	4	1.60	0.07	0.60
*Isopleth + log(UD)*	4	1.81	0.06	0.66
*Sex + Isopleth*	5	8.53	0.00	1.00

*UD*, utilization density; K, number of parameters; QAIC_c_Wt, QAIC_c_ weight; Cum.Wt, cumulative QAIC_c_ weight.

When overdispersion was handled with a GLMM, models containing a random effect for each animal camera combination were supported (Appendix S2). For Year 1, the simple models without and with the *Sex* effect are only 0.95 or 1.12 below the best model, suggesting only very weak evidence against the simple model. The coefficients for the *Isopleth* and *Core* effects in the best models indicate that animals in the *Core*, or low *Isopleth* intervals, tend to have slightly higher photo counts. For Year 2, the simple model (*1 + offset*[*log*(*UD*)]) was selected, again suggesting that no information beyond the *UD* values contributes to camera trap detection probabilities (Appendix S2).

**Figure 4:**
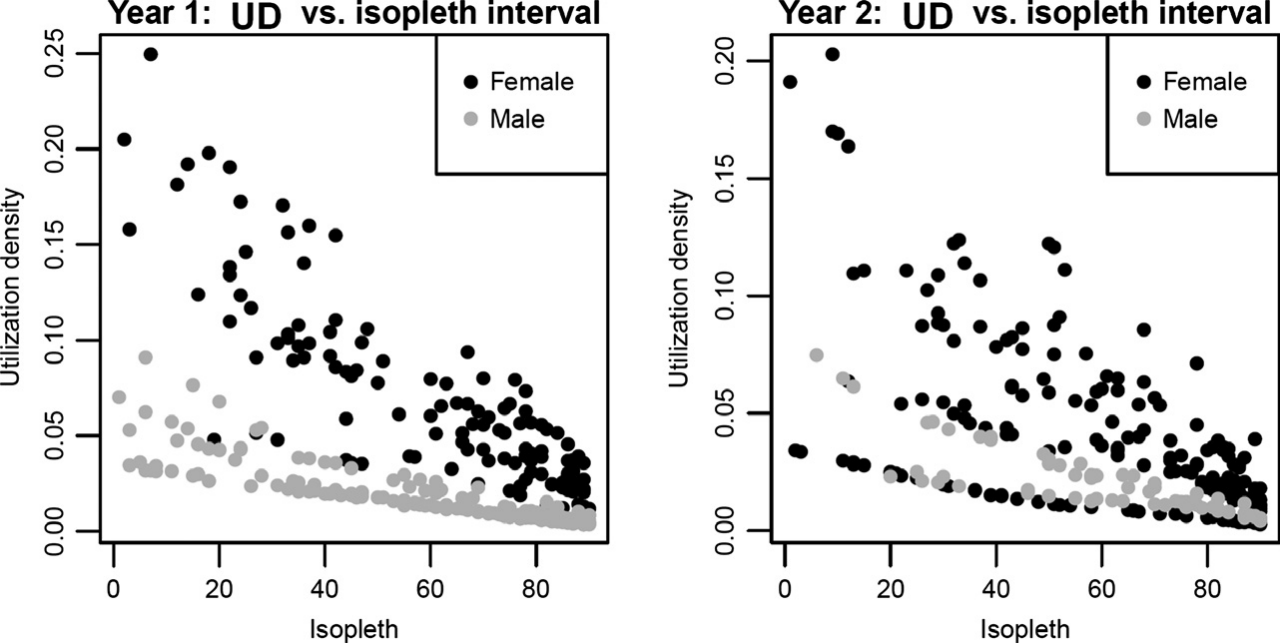
Data showing how heterogeneity in home range sizes generates variation in the relationship between the utilization density (*y*-axis) and the isopleth percentile (*x*-axis). For example, the utilization density of one male in Year 1 at its 60% isopleth is similar to that of the 30% isopleth for another male.

Models without log(*UD*), and relying only on *Isopleth*, *Core,* and/or *Sex,* had much worse fits (Tables 4, Appendix S2). This supports the hypothesis that camera trap probabilities are related to home ranges via the density of space use, as measured by the utilization density. Due to the large heterogeneity in home range sizes, utilization density is not precisely related to the isopleth percentile (Fig. 4), which explains how one variable can provide much better fits to camera trap data than the other.

#### Relations between camera captures and isopleth interval area

For both sexes, the mean number of telemetry relocations within cameras that successfully detected animals increases with utilization density, which is consistent with higher density space use toward the core of home ranges (Fig. S1). However, when males or females have one telemetry location within 500 m of a camera, the males tend to be in locations with a much smaller utilization density (Fig. 2, Fig. S1), that is, they are using space more sparsely by traversing larger areas. For Year 2, male detections still occurred at lower utilization densities, but the 95% CI around the mean isopleth interval areas overlapped for males and females (consistent with proximity results for Year 2).

## Discussion

Our results provide fundamental support for use of camera trap data to estimate animal space use by demonstrating that such data are largely consistent with more detailed telemetry data in the case of fishers in the Sierra Nevada. Although spatially explicit capture–recapture models assume simpler home range shapes than models for telemetry data, it is reasonable to conclude that camera trap data do reflect space use in a manner consistent with telemetry data. More generally, the methods we have provided can be applied to other systems where both camera trap and telemetry data are available. However, looking beyond that central conclusion, our findings also highlight several cautions for use of camera trap data only and make the case for integrating other sources of data, such as telemetry (e.g., Ivan et al. 2013; Royle et al. 2013; Sollmann et al. 2013a,b2013b).

In addition to heterogeneity inherent in camera trapping, our results suggest that there may be differences between years and between seasons within years in the relationship between movement and camera detection. The proximity results for Year 1 supported differences between sexes in attraction to nearby cameras, while Year 2 results showed seasonal differences. The latter finding was consistent with the hypothesis that animals are more likely to be captured at baited cameras during winter, when food is scarce (Fig. 2). Results from the home range analysis were more consistent between years (Fig. 3). Taken together, these results highlight the importance of not assuming that camera efficacy is consistent across seasons.

While our results supported the simple model that camera detection probability is proportional to *UD*, we did find weak evidence for more complicated relationships. Depending on the analysis model, there was weak evidence that animals tend to make repeated visits to cameras in the center of their home ranges more frequently than in the periphery. For females, the estimated relationship between log(*UD*) and photo counts was nearly identical to the simple model (Fig. 3D), but for males, the more complicated model explains a few high counts in low isopleth intervals (i.e., toward the home range core). Because the nature of those statistical results involves failing to reject the simple model in favor of more complicated ones (e.g., including variables *Core*, *Isopleth*, and *Sex*), it is worth continuing to consider such hypotheses for future studies.

How do the two analyses fit together? Our results demonstrate clearly the relationships between sex-specific home range sizes, attraction to cameras, and detection probabilities for a camera at a particular space-use density (*UD*). The proximity analysis revealed that, given the same number of nearby relocations, females were more likely to be captured at camera traps (during Year 1). The home range analysis suggested that, given the same size of an isopleth interval, males were more detectable during both years. These two main findings may appear contradictory until differences in home range sizes are considered (Table 1). For a given number of telemetry relocations per camera per individual, such relocations occur at lower *UD* values for males compared to females (Fig. S1). This is due to males having larger home ranges; thus, the time spent per unit area is less for males compared to females. For example, both males and females may spend the same amount of time in an area encompassing the 40–50% isopleth interval, but the size of that area for males may be five times larger than that of females. When males and females are detected within 250 m of a camera just once, males tend to be in area with lower *UD* values, hence are less likely to find the nearby camera. At locations with the same *UD*, however, males were more likely to find cameras due to higher rates of movement (males: 2339 ± 208 m/day; females: 1591 ± 72 m/day (mean ± 1 SE); R. Sweitzer, unpubl. data). The relationships between these variables further illuminate the types of processes that call for sex-specific detection probabilities in spatially explicit capture–recapture models.

In conclusion, our research represents the first empirical test for reconciling space use by animals using data gathered simultaneously using different sampling techniques and provides support for using camera traps for estimating space use with some cautions. Beyond space use, such data can be used in many other ways to examine aspects of animal behavior and management. For example, future studies could investigate (1) the effectiveness of camera traps at detecting all animals, whose home ranges overlap a particular camera trap station, and what level of camera trapping effort is required to do so and (2) animal density using a combination of mark–recapture (camera trap data) and telemetry data in a spatial capture–recapture framework.

## Supporting Information

Figure S1 Reconciling proximity-to-cameras and home range analysis.

Appendix S1 Model selection tables for the proximity analysis.

Appendix S2 Model selection tables for the home range analysis.
