# Supplementary material for: Testing the consistency of wildlife data types before combining them: the case of camera traps and telemetry
Source: Ecol Evol. 2014 Feb 24;4(7):933–43. doi: 10.1002/ece3.997 (PMC3997311; doi:10.1002/ece3.997)

**Figure S1.** Reconciling Proximity-to-cameras and Home range analysis: sex-specific differences in telemetry relocations within 250 m (a, c) and 500 m (b, d) from camera traps which successfully detected Pacific fishers vs. the mean utilization density (*UD*) and 95% CI intervals where relocations occurred.


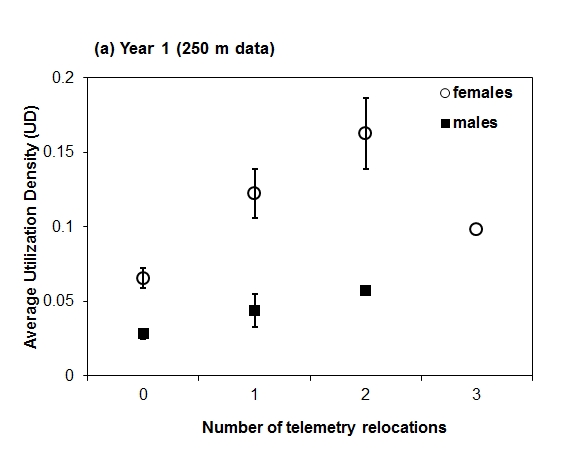

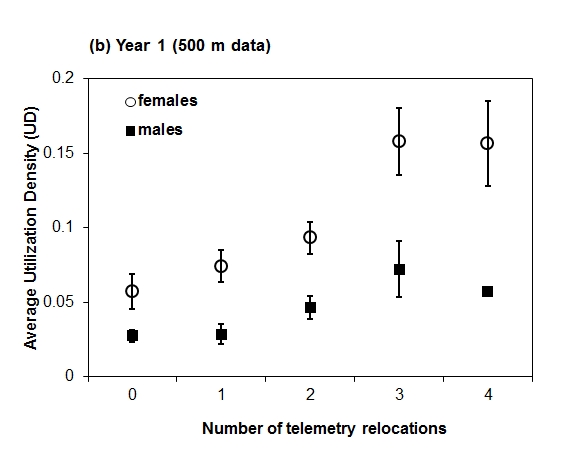

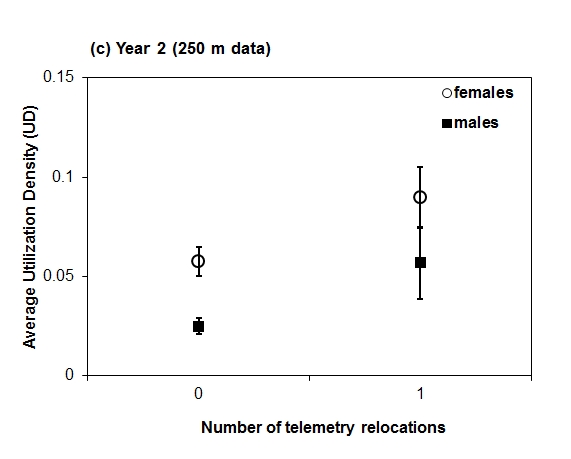

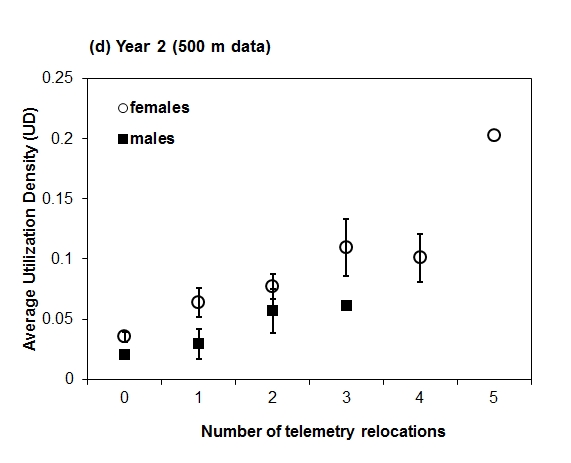

Supplement: Figure S1 — Reconciling proximity-to-cameras and home range analysis. [file ece30004-0933-sd1.docx]
