# Supplementary material for: Testing the consistency of wildlife data types before combining them: the case of camera traps and telemetry
Source: Ecol Evol. 2014 Feb 24;4(7):933–43. doi: 10.1002/ece3.997 (PMC3997311; doi:10.1002/ece3.997)
Supplement: Appendix S1 — Model selection tables for the proximity analysis. [file ece30004-0933-sd2.docx]

**Appendix S1. Model selection tables for the Proximity analysis**

**Table S1.1.** Proximity analysis results for Year 1 using binary GLMM (each model contains a random effect [intercept] for each Fisher); AICc for best models are 492.79 (250 m data), and 466.48 (500 m data). R^2^_GLMM_ represents the conditional R^2^ for general linear mixed-effects models developed by [Nakagawa & Schielzeth (2013](#_ENREF_1)).

| **Model** | **K** | **ΔAICc** | **AICcWt** | **CumWt** | **LL** | **R^2^** |
| --- | --- | --- | --- | --- | --- | --- |
| ***250 m data*** |  |  |  |  |  |  |
| Sex×Season + Locs250 | 6 | 0 | 0.39 | 0.39 | -240.32 | 0.24 |
| Sex+Season + Locs250 | 5 | 0.83 | 0.26 | 0.65 | -241.75 | 0.20 |
| Sex×Locs250 | 5 | 2.37 | 0.12 | 0.77 | -242.52 | 0.20 |
| Sex+Locs250 | 4 | 2.42 | 0.12 | 0.88 | -243.57 | 0.20 |
| Sex×Season×Locs250 | 9 | 2.49 | 0.11 | 0.99 | -238.47 | 0.27 |
| Season + Locs250 | 4 | 9.25 | 0 | 1 | -246.98 | 0.20 |
| Season×Locs250 | 5 | 10.74 | 0 | 1 | -246.71 | 0.21 |
|  |  |  |  |  |  |  |
| ***500 m data*** |  |  |  |  |  |  |
| Sex×Season×Locs500 | 9 | 0 | 0.82 | 0.82 | -224.07 | 0.42 |
| Season×Locs500 | 5 | 5.78 | 0.05 | 0.86 | -231.07 | 0.31 |
| Sex+Season + Locs500 | 5 | 5.85 | 0.04 | 0.91 | -231.11 | 0.27 |
| Sex×Season + Locs500 | 6 | 6.52 | 0.03 | 0.94 | -230.42 | 0.29 |
| Sex×Locs500 | 5 | 6.62 | 0.03 | 0.97 | -231.49 | 0.27 |
| Season + Locs500 | 4 | 7 | 0.02 | 0.99 | -232.70 | 0.27 |
| Sex+Locs500 | 4 | 9.23 | 0.01 | 1 | -233.82 | 0.27 |

**Table S1.2.** Proximity analysis results for Year 2 using binary GLMM (each model contains a random effect [intercept] for each Fisher); AICc for best models are 400.22 (250 m data), and 382.91 (500 m data).

| **Model** | **K** | **ΔAICc** | **AICcWt** | **CumWt** | **LL** | **R^2^** |
| --- | --- | --- | --- | --- | --- | --- |
| ***250 m data*** |  |  |  |  |  |  |
| Season+Locs250 | 4 | 0 | 0.48 | 0.48 | -196.06 | 0.13 |
| Season×Locs250 | 5 | 1.53 | 0.22 | 0.7 | -195.79 | 0.13 |
| Sex+Season+Locs250 | 5 | 1.79 | 0.19 | 0.9 | -195.93 | 0.14 |
| Sex×Season+Locs250 | 6 | 3.72 | 0.07 | 0.97 | -195.86 | 0.14 |
| Sex+Locs250 | 4 | 6.72 | 0.02 | 0.99 | -199.42 | 0.15 |
| Sex×Season×Locs250 | 9 | 8.15 | 0.01 | 0.99 | -194.94 | 0.24 |
| Sex×Locs250 | 5 | 8.75 | 0.01 | 1 | -199.40 | 0.15 |
|  |  |  |  |  |  |  |
| ***500 m data*** |  |  |  |  |  |  |
| Season+Locs500 | 4 | 0 | 0.4 | 0.4 | -187.4 | 0.18 |
| Season×Locs500 | 5 | 0.15 | 0.37 | 0.77 | -186.45 | 0.17 |
| Sex+Season+Locs500 | 5 | 2.05 | 0.14 | 0.91 | -187.40 | 0.18 |
| Sex×Season+Locs500 | 6 | 3.84 | 0.06 | 0.97 | -187.26 | 0.17 |
| Sex×Locs500 | 5 | 6.57 | 0.01 | 0.98 | -189.66 | 0.18 |
| Sex×Season×Locs500 | 9 | 7.36 | 0.01 | 0.99 | -185.88 | 0.18 |
| Sex+Locs500 | 4 | 7.45 | 0.01 | 1 | -191.12 | 0.17 |

**References**

Nakagawa, S. & Schielzeth, H. (2013) A general and simple method for obtaining R2 from generalized linear mixed-effects models. *Methods in Ecology and Evolution,* **4,** 133-142.
