# Supplementary material for: Testing the consistency of wildlife data types before combining them: the case of camera traps and telemetry
Source: Ecol Evol. 2014 Feb 24;4(7):933–43. doi: 10.1002/ece3.997 (PMC3997311; doi:10.1002/ece3.997)
Supplement: Appendix S2 — Model selection tables for the home range analysis. [file ece30004-0933-sd3.docx]

**Appendix S2. Model selection tables for the Home range analysis** (Tables S2.1-S2.2: Generalized Linear Models for binary data; Tables S2.3-S2.4: Generalized Linear Models for count data; Tables S2.5-S2.6: Generalized Linear Mixed Effects Models for count data)

**Table S2.1.** Model selection results for binary data GLMs, *Fall/Winter* Year 1. The overdispersion parameter for a GLM quasibinomial full model [*Sex* × *Core* × *log(1/Area)*)] was 1.01, which warranted the use of a regular binomial response and AICc for model selection instead of QAIC. K = number of parameters, AICcWt = Akaike weight, LL = log-likelihood, CumAICcWt = cumulative Akaike weight.

| **Model** | **K** | **ΔAICc** | **AICcWt** | **LL** | **CumAICcWt** |
| --- | --- | --- | --- | --- | --- |
| Sex + offset(log(UD)) | 2 | 0.00 | 0.18 | -146.73 | 0.18 |
| Sex × log(UD) | 4 | 0.25 | 0.16 | -144.81 | 0.35 |
| Sex × Core + offset(log(UD)) | 4 | 0.85 | 0.12 | -145.11 | 0.47 |
| Sex × Isopleth + offset(log(UD)) | 4 | 1.94 | 0.07 | -145.65 | 0.54 |
| Sex + Isopleth + offset(log(UD)) | 3 | 2.02 | 0.07 | -146.72 | 0.61 |
| Sex + log(UD) | 3 | 2.02 | 0.07 | -146.72 | 0.67 |
| Sex + Core + offset(log(UD)) | 3 | 2.04 | 0.07 | -146.73 | 0.74 |
| Core × log(UD) | 4 | 2.52 | 0.05 | -145.94 | 0.79 |
| Sex × Core + log(UD) | 5 | 2.83 | 0.04 | -145.07 | 0.84 |
| Isopleth + log(UD) | 3 | 3.72 | 0.03 | -147.57 | 0.87 |
| Sex × Isopleth + log(UD) | 5 | 3.99 | 0.03 | -145.64 | 0.89 |
| Sex + Core + log(UD) | 4 | 4.07 | 0.02 | -146.72 | 0.92 |
| Sex + Isopleth + log(UD) | 4 | 4.07 | 0.02 | -146.72 | 0.94 |
| Core + log(UD) | 3 | 4.60 | 0.02 | -148.01 | 0.96 |
| 1 + log(UD) | 2 | 4.62 | 0.02 | -149.04 | 0.98 |
| 1 + offset(log(UD)) | 1 | 7.04 | 0.01 | -151.26 | 0.98 |
| Sex × Isopleth × log(UD) | 8 | 7.11 | 0.01 | -144.06 | 0.99 |
| Sex × Core × log(UD) | 8 | 7.47 | 0.00 | -144.24 | 0.99 |
| Isopleth + offset(log(UD)) | 2 | 9.05 | 0.00 | -151.25 | 0.99 |
| Core + offset(log(UD)) | 2 | 9.06 | 0.00 | -151.26 | 1.00 |
| Sex × Isopleth | 4 | 9.30 | 0.00 | -149.33 | 1.00 |
| Sex + Isopleth | 3 | 9.98 | 0.00 | -150.70 | 1.00 |
| Sex × Core | 4 | 13.00 | 0.00 | -151.18 | 1.00 |
| Sex + Core | 3 | 15.55 | 0.00 | -153.48 | 1.00 |
| Isopleth | 2 | 15.88 | 0.00 | -154.67 | 1.00 |
| Core | 2 | 19.45 | 0.00 | -156.46 | 1.00 |
| Sex | 2 | 34.91 | 0.00 | -164.19 | 1.00 |

**Table S2.2.** Model selection results for binary data GLMs, *Fall/Winter* Year 2. The overdispersion parameter for a GLM quasibinomial full model [*Sex* × *Core* × *log(1/Area)*)] was 1.00, which warranted the use of a regular binomial response and AICc for model selection instead of QAIC. K = number of parameters, AICcWt = Akaike weight, LL = log-likelihood, CumAICcWt = cumulative Akaike weight.

| **Model** | **K** | **ΔAICc** | **AICcWt** | **LL** | **CumAICcWt** |
| --- | --- | --- | --- | --- | --- |
| Sex + Core + offset(log(UD)) | 3 | 0.00 | 0.14 | -130.19 | 0.14 |
| Sex + offset(log(UD)) | 2 | 0.10 | 0.13 | -131.26 | 0.28 |
| Sex + log(UD) | 3 | 0.54 | 0.11 | -130.46 | 0.38 |
| Sex + Isopleth + offset(log(UD)) | 3 | 0.79 | 0.10 | -130.58 | 0.48 |
| Sex × Isopleth + offset(log(UD)) | 4 | 1.50 | 0.07 | -129.90 | 0.55 |
| 1 + log(UD) | 2 | 1.62 | 0.06 | -132.02 | 0.61 |
| Sex × Core + offset(log(UD)) | 4 | 1.67 | 0.06 | -129.99 | 0.67 |
| Sex × log(UD) | 4 | 1.68 | 0.06 | -129.99 | 0.73 |
| Sex + Core + log(UD) | 4 | 1.78 | 0.06 | -130.04 | 0.79 |
| Sex + Isopleth + log(UD) | 4 | 2.47 | 0.04 | -130.39 | 0.83 |
| Sex × Isopleth + log(UD) | 5 | 3.10 | 0.03 | -129.67 | 0.86 |
| Core + log(UD) | 3 | 3.38 | 0.03 | -131.88 | 0.89 |
| Sex × Core + log(UD) | 5 | 3.45 | 0.03 | -129.84 | 0.91 |
| Isopleth + log(UD) | 3 | 3.65 | 0.02 | -132.01 | 0.94 |
| Core + offset(log(UD)) | 2 | 3.95 | 0.02 | -133.19 | 0.95 |
| 1 + offset(log(UD)) | 1 | 4.55 | 0.01 | -134.50 | 0.97 |
| Isopleth + offset(log(UD)) | 2 | 4.75 | 0.01 | -133.58 | 0.98 |
| Core × log(UD) | 4 | 4.99 | 0.01 | -131.65 | 0.99 |
| Sex × Isopleth × log(UD) | 8 | 7.72 | 0.00 | -128.80 | 1.00 |
| Sex × Core × log(UD) | 8 | 7.90 | 0.00 | -128.89 | 1.00 |
| Isopleth | 2 | 14.32 | 0.00 | -138.37 | 1.00 |
| Sex + Isopleth | 3 | 16.33 | 0.00 | -138.35 | 1.00 |
| Sex × Isopleth | 4 | 16.42 | 0.00 | -137.37 | 1.00 |
| Core | 2 | 20.53 | 0.00 | -141.47 | 1.00 |
| Sex + Core | 3 | 22.57 | 0.00 | -141.47 | 1.00 |
| Sex × Core | 4 | 24.21 | 0.00 | -141.26 | 1.00 |
| Sex | 2 | 24.91 | 0.00 | -143.66 | 1.00 |

**Table S2.3.** Model selection results for count data GLMs, *Fall/Winter* Year 1. The overdispersion parameter of 2.50 was estimated for the saturated fixed effects model [*Gender×Core×log(1/Area)*]. K = number of parameters, AICcWt = Akaike weight, QuasiLL = quasi log-likelihood, CumQAICcWt = cumulative Akaike weight.

| **Model** | **K** | **ΔQAICc** | **QAICcWt** | **QuasiLL** | **c_hat** | **CumQAICcWt** |
| --- | --- | --- | --- | --- | --- | --- |
| Sex + offset(log(UD)) | 3 | 0.00 | 0.15 | -131.65 | 2.50 | 0.15 |
| Sex × Core + offset(log(UD)) | 5 | 0.22 | 0.14 | -129.70 | 2.50 | 0.29 |
| Sex + Isopleth + offset(log(UD)) | 4 | 1.23 | 0.08 | -131.24 | 2.50 | 0.38 |
| Sex × log(UD) | 5 | 1.30 | 0.08 | -130.24 | 2.50 | 0.46 |
| Sex + log(UD) | 4 | 1.52 | 0.07 | -131.38 | 2.50 | 0.53 |
| Sex + Core + offset(log(UD)) | 4 | 1.60 | 0.07 | -131.42 | 2.50 | 0.60 |
| Isopleth + log(UD) | 4 | 1.81 | 0.06 | -131.53 | 2.50 | 0.66 |
| Sex × Core + log(UD) | 6 | 2.28 | 0.05 | -129.69 | 2.50 | 0.71 |
| 1 + offset(log(UD)) | 2 | 2.43 | 0.05 | -133.88 | 2.50 | 0.75 |
| Sex × Isopleth + offset(log(UD)) | 5 | 2.48 | 0.04 | -130.83 | 2.50 | 0.80 |
| Isopleth + offset(log(UD)) | 3 | 3.15 | 0.03 | -133.22 | 2.50 | 0.83 |
| Sex + Isopleth + log(UD) | 5 | 3.28 | 0.03 | -131.23 | 2.50 | 0.86 |
| Sex + Core + log(UD) | 5 | 3.56 | 0.03 | -131.37 | 2.50 | 0.89 |
| Core + log(UD) | 4 | 3.79 | 0.02 | -132.52 | 2.50 | 0.91 |
| 1 + log(UD) | 3 | 3.82 | 0.02 | -133.56 | 2.50 | 0.93 |
| Core + offset(log(UD)) | 3 | 3.93 | 0.02 | -133.62 | 2.50 | 0.95 |
| Core × log(UD) | 5 | 3.94 | 0.02 | -131.56 | 2.50 | 0.98 |
| Sex × Isopleth + log(UD) | 6 | 4.50 | 0.02 | -130.80 | 2.50 | 0.99 |
| Sex × Core × log(UD) | 9 | 8.41 | 0.00 | -129.58 | 2.50 | 0.99 |
| Sex × Isopleth × log(UD) | 9 | 8.50 | 0.00 | -129.63 | 2.50 | 1.00 |
| Sex + Isopleth | 4 | 8.53 | 0.00 | -134.89 | 2.50 | 1.00 |
| Sex × Isopleth | 5 | 9.26 | 0.00 | -134.22 | 2.50 | 1.00 |
| Sex × Core | 5 | 15.77 | 0.00 | -137.47 | 2.50 | 1.00 |
| Sex + Core | 4 | 18.90 | 0.00 | -140.07 | 2.50 | 1.00 |
| Isopleth | 3 | 20.16 | 0.00 | -141.73 | 2.50 | 1.00 |
| Core | 3 | 27.13 | 0.00 | -145.22 | 2.50 | 1.00 |
| Sex | 3 | 45.23 | 0.00 | -154.27 | 2.50 | 1.00 |

**Table S2.4.** Model selection results for count data GLMs, *Fall/Winter* Year 2. The overdispersion parameter of 2.16 was estimated for the saturated fixed effects model [*Gender×Core×log(1/Area)*]. K = number of parameters, AICcWt = Akaike weight, QuasiLL = quasi log-likelihood, CumQAICcWt = cumulative Akaike weight.

| **Model** | **K** | **ΔQAICc** | **QAICcWt** | **QuasiLL** | **c_hat** | **CumQAICcWt** |
| --- | --- | --- | --- | --- | --- | --- |
| Sex × Isopleth + offset(log(UD)) | 5 | 0.00 | 0.35 | -122.31 | 2.16 | 0.35 |
| Sex × log(UD) | 5 | 1.11 | 0.20 | -122.87 | 2.16 | 0.55 |
| Sex × Isopleth + log(UD) | 6 | 2.01 | 0.13 | -122.27 | 2.16 | 0.67 |
| Sex + offset(log(UD)) | 3 | 3.15 | 0.07 | -125.96 | 2.16 | 0.75 |
| 1 + offset(log(UD)) | 2 | 4.59 | 0.04 | -127.70 | 2.16 | 0.78 |
| Sex + log(UD) | 4 | 4.82 | 0.03 | -125.76 | 2.16 | 0.81 |
| Sex + Isopleth + offset(log(UD)) | 4 | 5.04 | 0.03 | -125.87 | 2.16 | 0.84 |
| Sex + Core + offset(log(UD)) | 4 | 5.20 | 0.03 | -125.95 | 2.16 | 0.87 |
| Sex × Core + offset(log(UD)) | 5 | 5.21 | 0.03 | -124.92 | 2.16 | 0.89 |
| Sex × Isopleth × log(UD) | 9 | 6.38 | 0.01 | -121.25 | 2.16 | 0.91 |
| Isopleth + offset(log(UD)) | 3 | 6.52 | 0.01 | -127.64 | 2.16 | 0.92 |
| 1 + log(UD) | 3 | 6.59 | 0.01 | -127.68 | 2.16 | 0.93 |
| Core + offset(log(UD)) | 3 | 6.63 | 0.01 | -127.70 | 2.16 | 0.94 |
| Sex + Core + log(UD) | 5 | 6.81 | 0.01 | -125.72 | 2.16 | 0.96 |
| Sex + Isopleth + log(UD) | 5 | 6.90 | 0.01 | -125.76 | 2.16 | 0.97 |
| Sex × Core + log(UD) | 6 | 6.94 | 0.01 | -124.73 | 2.16 | 0.98 |
| Sex × Core × log(UD) | 9 | 7.05 | 0.01 | -121.59 | 2.16 | 0.99 |
| Isopleth + log(UD) | 4 | 8.23 | 0.01 | -127.47 | 2.16 | 0.99 |
| Core + log(UD) | 4 | 8.62 | 0.00 | -127.66 | 2.16 | 1.00 |
| Core × log(UD) | 5 | 10.57 | 0.00 | -127.60 | 2.16 | 1.00 |
| Sex × Isopleth | 5 | 26.44 | 0.00 | -135.53 | 2.16 | 1.00 |
| Isopleth | 3 | 31.51 | 0.00 | -140.14 | 2.16 | 1.00 |
| Sex + Isopleth | 4 | 33.31 | 0.00 | -140.01 | 2.16 | 1.00 |
| Core | 3 | 42.26 | 0.00 | -145.52 | 2.16 | 1.00 |
| Sex × Core | 5 | 43.39 | 0.00 | -144.00 | 2.16 | 1.00 |
| Sex + Core | 4 | 43.91 | 0.00 | -145.31 | 2.16 | 1.00 |
| Sex | 3 | 57.21 | 0.00 | -152.99 | 2.16 | 1.00 |

**Table S2.5.** Model selection results for count data GLMMs using *Fisher/Camera* random effects, *Fall/Winter* Year 1. K = number of parameters, AICWt = Akaike weight, LL = log-likelihood, CumAICWt = cumulative Akaike weight.

| **Model** | **K** | **ΔAIC** | **AICWt** | **LL** | **CumAICWt** |
| --- | --- | --- | --- | --- | --- |
| Sex × Core + offset(log(UD)) + (1\|Fisher/CameraID) | 6 | 0.00 | 0.11 | -169.75 | 0.11 |
| Isopleth + offset(log(UD)) + (1\|Fisher/CameraID) | 4 | 0.06 | 0.10 | -171.78 | 0.21 |
| Sex + log(UD) + (1\|Fisher/CameraID) | 5 | 0.60 | 0.08 | -171.05 | 0.29 |
| Core + offset(log(UD)) + (1\|Fisher/CameraID) | 4 | 0.61 | 0.08 | -172.05 | 0.37 |
| Sex × log(UD) + (1\|Fisher/CameraID) | 6 | 0.84 | 0.07 | -170.17 | 0.44 |
| Sex + Isopleth + offset(log(UD)) + (1\|Fisher/CameraID) | 5 | 0.95 | 0.07 | -171.22 | 0.51 |
| 1 + offset(log(UD)) + (1\|Fisher/CameraID) | 3 | 0.95 | 0.07 | -173.22 | 0.57 |
| Sex + offset(log(UD)) + (1\|Fisher/CameraID) | 4 | 1.12 | 0.06 | -172.31 | 0.63 |
| Sex + Core + offset(log(UD)) + (1\|Fisher/CameraID) | 5 | 1.23 | 0.06 | -171.36 | 0.69 |
| Sex × Core + log(UD) + (1\|Fisher/CameraID) | 7 | 1.57 | 0.05 | -169.53 | 0.74 |
| Isopleth + log(UD) + (1\|Fisher/CameraID) | 5 | 1.64 | 0.05 | -171.57 | 0.79 |
| Sex × Isopleth + offset(log(UD)) + (1\|Fisher/CameraID) | 6 | 1.98 | 0.04 | -170.74 | 0.83 |
| Core + log(UD) + (1\|Fisher/CameraID) | 5 | 2.42 | 0.03 | -171.96 | 0.86 |
| Core × log(UD) + (1\|Fisher/CameraID) | 6 | 2.49 | 0.03 | -170.99 | 0.89 |
| Sex + Core + log(UD) + (1\|Fisher/CameraID) | 6 | 2.53 | 0.03 | -171.01 | 0.92 |
| Sex + Isopleth + log(UD) + (1\|Fisher/CameraID) | 6 | 2.57 | 0.03 | -171.03 | 0.95 |
| 1 + log(UD) + (1\|Fisher/CameraID) | 4 | 2.78 | 0.03 | -173.14 | 0.98 |
| Sex × Isopleth + log(UD) + (1\|Fisher/CameraID) | 7 | 3.72 | 0.02 | -170.61 | 1.00 |
| Sex × Core × log(UD) + (1\|Fisher/CameraID) | 10 | 7.51 | 0.00 | -169.50 | 1.00 |
| Sex + Isopleth + (1\|Fisher/CameraID) | 5 | 8.92 | 0.00 | -175.21 | 1.00 |
| Sex × Isopleth + (1\|Fisher/CameraID) | 6 | 9.49 | 0.00 | -174.49 | 1.00 |
| Sex × Core + (1\|Fisher/CameraID) | 6 | 13.52 | 0.00 | -176.51 | 1.00 |
| Sex + Core + (1\|Fisher/CameraID) | 5 | 15.73 | 0.00 | -178.61 | 1.00 |
| Isopleth + (1\|Fisher/CameraID) | 4 | 17.87 | 0.00 | -180.68 | 1.00 |
| Core + (1\|Fisher/CameraID) | 4 | 22.22 | 0.00 | -182.86 | 1.00 |
| Sex + (1\|Fisher/CameraID) | 4 | 37.94 | 0.00 | -190.72 | 1.00 |

**Table S2.6.** Model selection results for count data GLMMs using *Fisher/Camera* random effects, *Fall/Winter* Year 2. K = number of parameters, AICWt = Akaike weight, LL = log-likelihood, CumAICWt = cumulative Akaike weight.

| **Modnames** | **K** | **ΔAIC** | **AICWt** | **LL** | **CumAICWt** |
| --- | --- | --- | --- | --- | --- |
| 1 + offset(log(UD)) + (1\|Fisher/CameraID) | 3 | 0.00 | 0.18 | -147.33 | 0.18 |
| Sex + offset(log(UD)) + (1\|Fisher/CameraID) | 4 | 0.99 | 0.11 | -146.83 | 0.29 |
| Sex × Isopleth + offset(log(UD)) + (1\|Fisher/CameraID) | 6 | 1.64 | 0.08 | -145.15 | 0.37 |
| Isopleth + offset(log(UD)) + (1\|Fisher/CameraID) | 4 | 1.77 | 0.08 | -147.22 | 0.45 |
| 1 + log(UD) + (1\|Fisher/CameraID) | 4 | 1.83 | 0.07 | -147.25 | 0.52 |
| Core + offset(log(UD)) + (1\|Fisher/CameraID) | 4 | 1.98 | 0.07 | -147.32 | 0.59 |
| Sex × log(UD) + (1\|Fisher/CameraID) | 6 | 2.13 | 0.06 | -145.40 | 0.65 |
| Sex + log(UD) + (1\|Fisher/CameraID) | 5 | 2.25 | 0.06 | -146.46 | 0.71 |
| Sex + Isopleth + offset(log(UD)) + (1\|Fisher/CameraID) | 5 | 2.73 | 0.05 | -146.70 | 0.76 |
| Sex + Core + offset(log(UD)) + (1\|Fisher/CameraID) | 5 | 2.98 | 0.04 | -146.82 | 0.80 |
| Sex × Isopleth + log(UD) + (1\|Fisher/CameraID) | 7 | 3.37 | 0.03 | -145.02 | 0.83 |
| Core + log(UD) + (1\|Fisher/CameraID) | 5 | 3.63 | 0.03 | -147.15 | 0.86 |
| Isopleth + log(UD) + (1\|Fisher/CameraID) | 5 | 3.75 | 0.03 | -147.21 | 0.89 |
| Sex + Core + log(UD) + (1\|Fisher/CameraID) | 6 | 3.78 | 0.03 | -146.23 | 0.92 |
| Sex × Core + offset(log(UD)) + (1\|Fisher/CameraID) | 6 | 3.88 | 0.03 | -146.27 | 0.95 |
| Sex + Isopleth + log(UD) + (1\|Fisher/CameraID) | 6 | 4.24 | 0.02 | -146.45 | 0.97 |
| Sex × Core + log(UD) + (1\|Fisher/CameraID) | 7 | 4.82 | 0.02 | -145.74 | 0.98 |
| Core × log(UD) + (1\|Fisher/CameraID) | 6 | 5.59 | 0.01 | -147.13 | 1.00 |
| Sex × Core × log(UD) + (1\|Fisher/CameraID) | 10 | 7.67 | 0.00 | -144.17 | 1.00 |
| Sex × Isopleth + (1\|Fisher/CameraID) | 6 | 12.75 | 0.00 | -150.71 | 1.00 |
| Isopleth + (1\|Fisher/CameraID) | 4 | 13.82 | 0.00 | -153.24 | 1.00 |
| Sex + Isopleth + (1\|Fisher/CameraID) | 5 | 14.56 | 0.00 | -152.61 | 1.00 |
| Core + (1\|Fisher/CameraID) | 4 | 24.18 | 0.00 | -158.42 | 1.00 |
| Sex + Core + (1\|Fisher/CameraID) | 5 | 25.00 | 0.00 | -157.83 | 1.00 |
| Sex × Core + (1\|Fisher/CameraID) | 6 | 25.71 | 0.00 | -157.19 | 1.00 |
| Sex + (1\|Fisher/CameraID) | 4 | 33.75 | 0.00 | -163.21 | 1.00 |
